# Complete chloroplast genome of Serrated Tussock, *Nassella trichotoma* (Poaceae: Stipeae)

**DOI:** 10.1080/23802359.2022.2107444

**Published:** 2022-08-04

**Authors:** Aisuo Wang, Hanwen Wu, David Gopurenko

**Affiliations:** NSW Department of Primary Industries, Wagga Wagga Agricultural Institute, PMB, Wagga Wagga, Australia

**Keywords:** Illumina sequencing, invasive grass, *Nassella*, chloroplast genome

## Abstract

*Nassella trichotoma* is one of the most serious weed species in Australia. It is often confused with other *Nassella* and stipoid species, especially at the young seedling stage, adding another layer of complexity in effective weed management. We report here the complete chloroplast genome of *N. trichotoma* (137,568 bp, GenBank accession number KX792500.2) sequenced using Next Generation Sequencing technology (Illumina). The *N. trichotoma* was grouped closely with other *Nassella* species and separated from other Stipeae species in the phylogenetic tree constructed based on the complete chloroplast genome sequences. The sequence information could be used for further identification of novel DNA barcodes for correct weed identification and subsequently improve management of this invasive grass.

*Nassella trichotoma* (Nees) Hack. ex Arechav 1896, commonly known as Serrated Tussock, is listed as a Weed of National Significance in Australia (McLaren et al. 2002). The invasive grass causes serious damage to the environment by displacing palatable grasses from pastures, decreasing the productivity of grazing livestock, and significantly degrading biodiversity in native grasslands. Effective management of this invasive weed relies on correct weed identification, particularly at the young seedling stage. Earlier attempts at genetic diagnostics of *N. trichotoma* have been hindered by a lack of informative loci to adequately distinguish it from several other stipoid grass species (Wang et al. 2014; Wang et al. 2017). We thus sequenced the complete chloroplast genome (cp) of *N. trichotoma*. We aimed to typify the genomic content of this *Nassella* species and determine if this will facilitate the identification of novel DNA barcode regions for species identification.

Illumina sequencing technique was employed to study the complete chloroplast genome of *N. trichotoma* using a specimen collected from Wagga Wagga NSW in Australia (34°58′ 40.7“, 147° 26′ 19.5“). The specimen was taxonomically identified as *N. trichotoma* and retained at Wagga Wagga Agricultural Institute (WWAI) under voucher number ww19856 (contact person: Dr Hanwen Wu, hanwen.wu@dpi.nsw.gov.au). Total genomic DNA was extracted from fresh leaves of *N. trichotoma* using CTAB protocol (Doyle and Doyle 1990). The extracted DNA was subject to library construction (500 bp insert size) and Illumina sequencing (125 PE) at Beijing Genomics Institute (BGI, Hong Kong).

The sequencing process generated a total of 13,324,374 raw reads, which gave more than 100-fold coverage of the chloroplast genome (SRA SRP345984, BioProject PRJNA780245, BioSamples SAMN23133359). Low quality reads, adapter sequences and duplications sequences in the raw reads were trimmed with readfq v5 (https://github.com/lh3/readfq). The clean reads (13,194,420) were de novo assembled with SOAPdenovo-Trans (Xie et al. 2014) (the kmer size was optimized to 61). GapCloser (Luo et al. 2012) was applied to fill the gaps between the scaffolds using default settings. Annotation of the *N. trichotoma* chloroplast genome was performed using CPGAVAS (Liu et al. 2012), which was then followed by subsequent check with DOGMA (Wyman et al. 2004) at default settings. The predicted annotations were verified using BLAST similarity search (Altschul et al. 1990).

All annotations were manually adjusted as needed before submission to GenBank (Accession No. KX792500.2). The complete cp genome of *N. trichotoma* (137,568 bp in length) was circular in shape, having a typical quadripartite structure (LSS, SS, IRa, IRb). It consisted of 89 protein coding genes, 39 tRNA genes and eight rRNA genes, similar to other published *Nassella* cp genomes. A total of 115 SNP sites were revealed between the cp genome sequences of *N. trichotoma* and the three published *Nassella* cp genomes. Most of these SNP sites were in the genes or regions of *matK* (23), *psbA* (12), *matK-rps16* intergenic spacer (12) and *psbA-matk* intergenic spacer (10).

A total of 27 complete chloroplast sequences for the tribe Stipeae were downloaded from GenBank (accessed on 6th August 2021). These sequences were aligned with the corresponding sequences of *N. trichotoma* and an outgroup *Triticum turgidum* (tribe Triticeae) (NC_024814.1) using MAFFT (Katoh et al. 2005) at the default settings. The resulting alignment file was applied to construct a Maximum Parsimony (MP) tree using the Subtree-Pruning-Regrafting (SPR) algorithm (Nei and Kumar 2000) in MEGA X (Kumar et al. 2018) (Figure 1). MP clade supports were obtained by 1,000 bootstrap replications.

**Figure 1.**
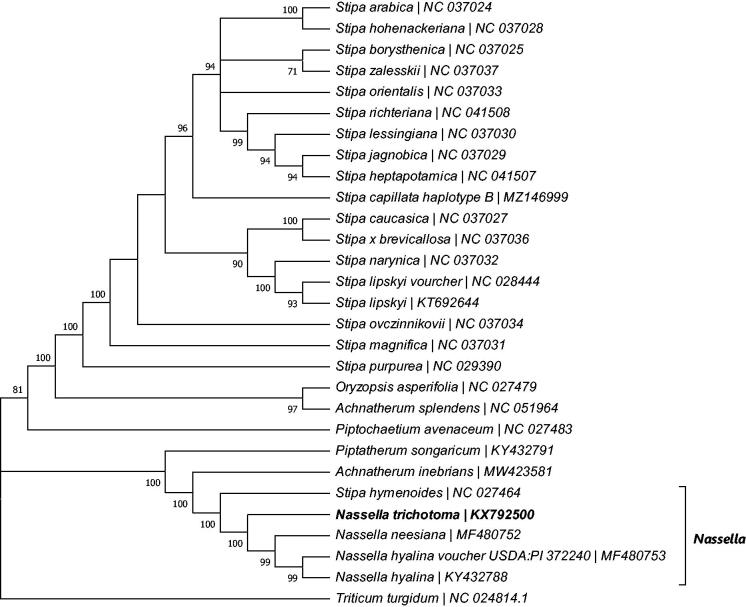
Maximum parsimony phylogenetic tree constructed on the complete chloroplast genomes of *N. trichotoma* and 27 other Stipeae species with *Triticum turgidum* as the outgroup.

The resulting MP phylogenetic tree of tribe Stipeae grouped *N. trichotoma* with other *Nassella* species (100 bootstrapping support) and separated all *Nassella* species from the remaining Stipa species. One of the Stipa species, *Stipa hymenoides* (NC027464), was placed near the *Nassella* clade, which is resulted from the low mismatch numbers (639) between the cp genome of *S. hymenoides* and *N. trichotoma* (the average mismatch numbers between the cp genomes of *N. trichotoma* and the remaining Stipa species is around 1300). The close phylogenetic relationship between *S. hymenoides* and the *Nassella* requires more research to verify. In summary, the complete chloroplast genome of *N. trichotoma* provides valuable information for further genetics studies (such as DNA barcoding, evolution, and phylogeny of *Nassella* species), thereby contributing to improved understanding and management of this weed.

## Data Availability

The genome sequence data that support the findings of this study are openly available in GenBank of NCBI at (https://www.ncbi.nlm.nih.gov/) under the accession no. KX792500.2 (The associated SRA number: SRP345984, BioProject number: PRJNA780245, BioSamples number: SAMN23133359).
